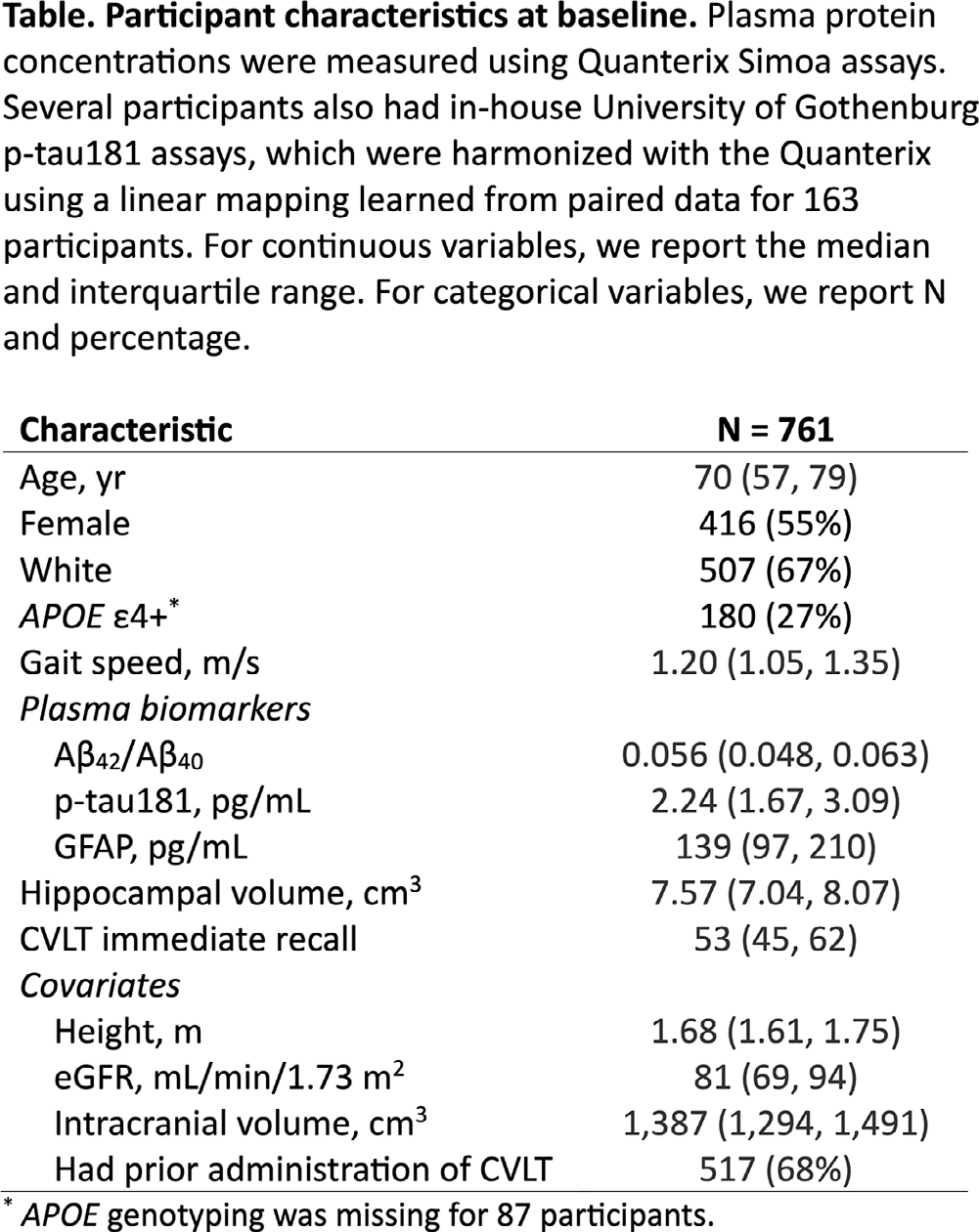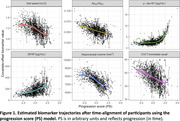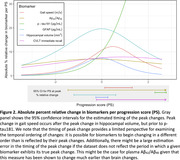# Temporal order of gait speed decline relative to changes in measures affected early in Alzheimer’s disease

**DOI:** 10.1002/alz.086001

**Published:** 2025-01-09

**Authors:** Murat Bilgel, Qu Tian, Keenan A. Walker, Abhay Moghekar, Nicholas J. Ashton, Przemyslaw Radoslaw Kac, Thomas K Karikari, Kaj Blennow, Henrik Zetterberg, Madhav Thambisetty, Susan M. Resnick

**Affiliations:** ^1^ National Institute on Aging, National Institutes of Health, Baltimore, MD USA; ^2^ National Institute on Aging, Baltimore, MD USA; ^3^ Laboratory of Behavioral Neuroscience, National Institute on Aging, Intramural Research Program, Baltimore, MD USA; ^4^ Johns Hopkins University School of Medicine, Baltimore, MD USA; ^5^ Centre for Age‐Related Medicine, Stavanger University Hospital, Stavanger Norway; ^6^ Department of Psychiatry and Neurochemistry, Institute of Neuroscience and Physiology, The Sahlgrenska Academy, University of Gothenburg, Mölndal, Gothenburg Sweden; ^7^ King’s College London, Institute of Psychiatry, Psychology & Neuroscience, Maurice Wohl Clinical Neuroscience Institute, London United Kingdom; ^8^ NIHR Biomedical Research Centre for Mental Health and Biomedical Research Unit for Dementia at South London and Maudsley, NHS Foundation, London United Kingdom; ^9^ Department of Psychiatry and Neurochemistry, Institute of Neuroscience and Physiology, The Sahlgrenska Academy at the University of Gothenburg, Mölndal Sweden; ^10^ Department of Psychiatry, School of Medicine, University of Pittsburgh, Pittsburgh, PA USA; ^11^ Department of Psychiatry and Neurochemistry, Institute of Neuroscience and Physiology, University of Gothenburg, Mölndal Sweden; ^12^ Clinical Neurochemistry Laboratory, Sahlgrenska University Hospital, Mölndal Sweden; ^13^ Hong Kong Center for Neurodegenerative Diseases, Hong Kong China; ^14^ Wisconsin Alzheimer’s Disease Research Center, University of Wisconsin School of Medicine and Public Health, Madison, WI USA; ^15^ Department of Psychiatry and Neurochemistry, Institute of Neuroscience and Physiology, the Sahlgrenska Academy at the University of Gothenburg, Mölndal Sweden; ^16^ UK Dementia Research Institute at UCL, London United Kingdom

## Abstract

**Background:**

Individuals with dual decline in gait and cognition have a greater risk of developing dementia. Understanding when gait speed declines relative to measures affected early in Alzheimer’s disease can improve risk assessment.

**Method:**

Using data for 761 participants (1,108 cognitively unimpaired visits) from the Baltimore Longitudinal Study of Aging, we estimated the trajectories of gait speed, memory (California Verbal Learning Test [CVLT] immediate recall score), hippocampal volume, and plasma Aβ_42_/Aβ_40_, glial fibrillary acidic protein (GFAP), and p‐tau181.

We fitted a progression score (PS) model that aligns individuals based on the similarity of their multivariate longitudinal observations to reveal long‐term biomarker trajectories, accounting for individual differences in baseline level and rate of progression. We included height as a covariate for gait speed, first administration versus not for CVLT (to control for practice effect), intracranial volume for hippocampal volume, and estimated glomerular filtration rate for each plasma biomarker.

74 participants converted to mild cognitive impairment (MCI) or dementia after the last visit used for fitting the PS model and were used to verify that the estimated PS reflects neurodegenerative disease progression using a Cox proportional hazards model. We then examined the temporal ordering of the estimated biomarker trajectories by comparing the timing of peak relative changes.

**Result:**

Greater PS at last visit was associated with a higher risk of conversion to MCI/dementia (hazard ratio for interquartile range normalized PS = 6.1, P < 2×10^‐16^). The PS‐only model had a better concordance index compared to an age‐only model for predicting conversion (0.880 ± 0.016 vs. 0.852 ± 0.018). Estimated trajectories as a function of PS are shown in Figure 1. The peak relative change in hippocampal volume occurred prior to that of gait speed (difference in PS between peaks: ‐0.57 [95% confidence interval ‐1.32, ‐0.05]), whereas p‐tau181 changed later (difference in PS between peaks: 1.95 [1.19, 2.45]) (Figure 2).

**Conclusion:**

Decline in gait speed occurs after hippocampal volume loss but alongside other biomarkers known to be affected early in Alzheimer’s disease and it can prove useful in individualized prediction. Alternative approaches for examining temporal ordering will help assess robustness of findings.